# Particulate Matter and Respiratory Symptoms among Adults Living in Windhoek, Namibia: A Cross Sectional Descriptive Study

**DOI:** 10.3390/ijerph14020110

**Published:** 2017-01-24

**Authors:** Ndinomholo Hamatui, Caryl Beynon

**Affiliations:** 1 Department of Health Sciences, Namibia University of Science and Technology, Private Bag 13388, Windhoek 9000, Namibia; 2 Laureate Online Education, University of Liverpool, Liverpool L69 3BX, UK; caryl.beynon@online.liverpool.ac.uk

**Keywords:** respiratory symptoms, PM exposure, residential location, Namibia, Windhoek

## Abstract

This study aimed to estimate the prevalence of respiratory symptoms and to assess respiratory health risks associated with Particulate Matter (PM) exposure among the residents of Windhoek, Namibia. **Objectives:** To measure particulate pollution concentration in Windhoek through monitoring of particulate matter concentration and to identify any associations between particulate pollution, individual location, and respiratory health among the Windhoek resident’s. **Methods:** an adapted standardized self-administered questionnaire was used to collect respiratory health related data as well as previous exposure, while PM monitoring was done using the ASTM (American Standard Test Method) D1739 reference method. **Results:** A high prevalence was observed for cough (43%), breathlessness (25%), and asthma (11.2%). PM was found to be a significant risk factor for episodes of coughing and phlegm, while high PM exposure category had an increased odds ratio (OR) for episodes of phlegm and cough (OR: 2.5, 95% CI (95% confidence intervals): 0.8–8.0). No association was observed between location and respiratory health outcomes. **Conclusions:** The study found high levels of PM concentration across all Windhoek suburbs which were above the German, American, and Environmental Protection Agency (EPA). Enactment of legislation relating to the control and monitoring of PM related emissions at the point of generation is required at both a country and city level.

## 1. Introduction 

Particulate Matter (PM) refers to a mixture of coarse and fine solid materials combined with liquid droplets; it has the ability to remain suspended in the air [1,2]. Particle pollutants are both primary and secondary in nature, with most primary particulate pollutants, especially the fine PM, originating from anthropogenic activities such as the combustion process at domestic and industrial settings [1,3]. PM remains a major public health concern globally, but its impact on population health in developing countries remains unclear. Health effects associated with PM exposure depends greatly on the PM size, morphology, elemental constituents, and frequency of exposure, which can be further influenced by individual socioeconomic status and lifestyle [1,2,4,5,6]. According to the World Health Organization (WHO), in 2012 the ambient air quality contributed to 3.7 billion premature deaths globally, accounting for 80% of premature deaths (defined as early end of individual life before expected age) [7]. Moreover, in 2014, WHO reported that, globally, 14% of Chronic Obstruction Pulmonary Diseases (COPD) related deaths and acute lower respiratory infections and 6% of lung cancer related deaths were attributed to poor air quality. Developing countries are reported to suffer most from particle pollution, probably due to the lack of control measures and lack of efforts to monitor and control pollution at the point of generation [7]. 

The evidence on the country (Namibia) burden of environmental related respiratory diseases has not been well established; the available literature is based on old data, reported as late as 2004. As of 2004, the country burden of diseases attributed to outdoor pollution was estimated to account for 10% of all deaths per year [7]. Also in 2009, WHO reported that Namibian prevalence rate for COPD and asthma was at 0.5 and 1.7 per 1000 people for the general population, respectively [8,9,10]. In 2004, the country (Namibia) mean for “particulate matter of 10 micrometers or less in diameter” (PM_10)_ in urban areas was reported at 50 μg/m^3^; this figure is estimated to have doubled since that time due to increased industrial activities as well increased vehicle emissions [7,8]. 

Although there is sufficient evidence to confirm the association between PM and poor health outcomes, such evidence is limited to studies conducted in developed countries whose pollution levels and sources are different to that of Namibia, mainly due to the level and type of industries in such countries. Moreover, the country lacks up-to-date data on particle pollution as well as associated health effects. Although the country level of industries is relatively small, considering the country’s new developmental agenda, establishing current pollution levels as well as related health effects is necessary to provide evidence to inform public health policy geared toward particulate pollution control and monitoring.

## 2. Study Sites

The study was conducted in Windhoek, the capital city of Namibia. Windhoek was considered a suitable setting due to the level of industries and motor vehicles which are key contributors to particulate pollution. According to the World Bank surveyed conducted in 2009, 103 persons per 1000 Namibians owned cars [11]. Further, it is estimated that 13% of the Windhoek residents own cars, while the remaining 87% use public transport, which includes city buses and taxis [12]. Windhoek is situated in the central region of the country, at an elevation of 1700 m above sea level, with a total space of 645 km^2^. The city has a population of about 325,858 people, with a growth rate of 3.9 and a population density of 9.2 persons/km^2^ [13]. The population composition is made up of 11% of children under five years old, 16% of 5–14 age group, and 69% of 15–59 age group, while those falling in the age category of 60 and above makes up only 4% of the city population [13]. The city accommodates 89,438 households with an average size of 3.7 persons per household. Thirty-nine percent of households are headed by females. In addition to that, 1% of city households do not have access to potable and sanitary facilities, while 8% of households use wood and charcoal for cooking and for other energy requirement purposes [13].

## 3. Methodology

### 3.1. Study Design 

The study employed non experimental approach; a descriptive cross sectional study was used, using a quantitative approach to data collection and analysis. This type of study design allows data collection at a single point in time, allowing both the measurement of disease prevalence and PM concentration at the same time [14]. 

### 3.2. Participants Selection and Data Collection Procedures 

The study populations were residents of Windhoek, who have lived in the city for a minimum of two years and are residents of any of the suburbs in which dust monitoring stations were placed. A total of 107 Windhoek residents who met inclusion criterial and agreed to take part participated in the study. The study was conducted between July 2015 and March 2016. 

#### 3.2.1. Sampling Approach

The study used a systematic random sampling approach to select households. One adult was selected per selected household based upon convenience. The number of households selected was proportional to the size of the suburb. 

#### 3.2.2. Sampling Frame

Windhoek has 11 suburbs (constituencies) including one rural; to obtain the final sample size, the researcher used the 2011 country census frame to draw the sample, using the number of households per constituency.

#### 3.2.3. Recruitment

The potential participants were identified and recruited using house location from a map in the city of Windhoek. The city map was used to identify the first respondent’s household, subsequent respondents were recruited using a pre-determined systematic approach of selecting every 5th house in the street situated near the PM sampling location, in cases of a locked house or if the identified household would not participate, the house immediately following was selected instead. The sequence was followed until all required respondents were reached per suburb. 

Following sampling and identification of the household; the researcher approached the prospective respondent, presenting basic information on the purpose of the visit to the house. Subsequently, a participant information sheet (PIS) with details on the research project was presented if the adult was found to have met recruitment criteria; after giving the PIS, the researcher spent maximum of 30 min with the prospective respondent to allow him/her to ask questions and for the researcher to answer questions.

#### 3.2.4. Data Collection Methods

##### Questionnaire Data

An adapted standardized self-administered questionnaire was used to collect data on respondents’ respiratory symptoms and diseases; family history of respiratory diseases; occupational exposure and history; and type of fuel used for heating and cooking. The following adaptions were made: respondent’s house distance from main road, type of road surface for main street/road passing by the house, years spent at current house as well as total years that respondents had lived in Windhoek (WHK). The adapted questionnaire was previously used to collect data related to respiratory health outcomes among cola workers in the US and South Africa [15]. Similarly, the questionnaire was translated into Oshiwambo and Afrikaans and translated back to English to ensure consistency. The completed questionnaires were posted back to the researcher using an envelope provided with the questionnaire.

##### Pilot-Testing

The questionnaire was first tested in one suburb using a sample of ten residents to test for consistency in questions and responses [16]. The respondents were asked to complete the questionnaire, which were then assessed for consistency. From the observation of data obtained, the researcher found no technical issues or ambiguous questions, all respondent’s answers were consistent, and no clarity was sought with reference to questions asked in the questionnaire.

##### PM Concentration Monitoring Data

The ASTM D1739 reference method was used for the collection of PM concentration level data and for final calculation of PM concentration levels to determine agreement between the PM concentrations received over the sampling period [17]. Particulate monitoring was conducted over a period of three months. The buckets were left open for a period of 30 days for each sampling month. The ordinary buckets with surface area of 0.043 m^2^ were placed at various points across the selected residential areas, at a pole height of 2 m and were left open, collecting dust irrespective of wind direction. The buckets were filled with 5 L of distilled water and hydrogen peroxide was added to prevent algae growth. Dust samples were transported to the Namibia University of Science and Technology, Environmental Health sciences laboratory, where the dust was extracted gravimetrically using the Buchner funnel apparatus. Filter papers used in the gravimetric analysis of collected dust were weighed both before and after filtration using a weighing scale with six digits. The final PM concentration levels were calculated using the following formula: Fall-out rate (mg/m^2^/day) = (collected mass × 1)/(0.043 × days).

Monthly average PM level was calculated and allocated to individuals living within 1 km of the particular station. 

### 3.3. Ethical Consideration and Informed Consent 

The respondents were briefed in person about the study and given a participant information sheet describing the purposes of research. The questionnaire was left with the person and they completed it if they wished to. By completing the questionnaire, they implied consent. Codes were used instead of participant’s names to ensure anonymity; to ensure confidentiality, questionnaires were stored in a lockable and secured cabinet at the researcher’s office at the Namibia University of Science and Technology. In addition to that, all related documents were stored in a password protected file. The ethical approval was obtained from the ethics committee of the University of Liverpool and the Ministry of Health and Social Services (Namibia) Ethics Committee (ref:17/3/3). 

### 3.4. Data Analysis 

The obtained data were analyzed using the statistical package for social scientist (SPSS) software version 21 (SPSS Inc., Chicago, IL, USA). The data was first cleaned through visual inspection as well as by means of basic descriptive analysis to look for odd values as well as characters not meant to be in the results. Explorative tests were done to test for data normality distribution using graphic outputs such as histograms and boxplots to detect outliers. This was followed by descriptive statistical analysis for both continuous and categorical variables. Following the explorative tests for normality, the continuous variables such as age, distance from the road, number of cigarettes smoked, and number of years spent at current home were described using mean and standard deviation, while skewed data were presented as median instead of mean. Categorical variables were analyzed and presented using counts and percentages, followed by a group difference test for categorical variables (chi-square and cross tabulation); descriptive test results were summarized using frequency tables. To allow for group difference analysis, continuous data were recorded in groups using tertiles, such variable’s included age, distance from road, and years at current home. PM concentration was a variable that was analyzed; it was defined as low, medium, or high based on tertile measurements. Outcome variables included a response that the respondent had respiratory symptoms and diseases, of which expected answers were a binary type of data (yes or no), which was then used in the test of differences using a chi-square test as deemed appropriate. In addition to that, the overall prevalence of respiratory symptoms was calculated and also described in terms of individual location, PM levels and previous exposures. Continuous variables collected included age, PM levels, and length of stay at current house. Categorical variables gathered included location, education level, and respiratory symptoms among others. Moreover, logistic regression was used to estimate the odds ratio with 95% confidence intervals (95% CI) of health outcomes. Multivariate logistic regression was used to test for associations between variables of interest. Significance test was accepted at *p* < 0.05. Possible confounding factors collected through the questionnaire included: age, gender, tuberculosis (TB), smoking, occupational exposure, asthma, and type of fuel used. The analyses were adjusted for these confounding factors. Both gender, TB, smoking, occupational exposure, asthma, and type of fuel used were obtained as categorical in nature, while Age was obtained a as discrete, continuous variable.

## 4. Results

### 4.1. Study Population Characteristics

Descriptive statistics were performed and the summary of the sample population’s general characteristics is described in Table 1. The majority of respondents fell in the age group of less than 30 years. Similarly, the majority of respondents were unmarried (73.8%), only a small fraction of the respondents reported that they smoked cigarettes (7.5%), and there was no difference between the proportion of ex- smokers and current smokers in the studied population. 

### 4.2. History on Environmental Related PM Exposure

Table 2 describes the studied population history of PM exposure, which illustrates that 22.4% of respondents live in areas with unsurfaced main streets/roads, while only 11% of respondents reported to have worked in environments which exposed them to dust for 1 year or more. Similarly, only 63.5% of respondents used electricity as a source of energy for cooking while the rest use carbon fuel for cooking such as gas, wood, and paraffin. The majority of respondents reported constant (43.9%) or frequent (39.3%) vehicle movement near their home or indoor dust observed mainly on surfaces (42.1%).

### 4.3. PM Concentration Levels across Suburbs

The results for PM measurement are presented in Table 3. High levels of particulate pollution were recorded across all suburbs regardless of the residential area classification for both monitoring periods; with the majority of dust levels recorded exceeding the recommend levels for residential areas by the American Standard Test Method, ASTM D1739. Suburbs in high density areas recorded the highest PM levels for all three monitoring periods. 

The Analysis of Variance (ANOVA) test showed a statistically significant difference in mean PM concentration levels between the low density and high density area (*p* = 0.002) (Table 4).

### 4.4. Prevalence of Respiratory Symptoms

One of the study objectives was to determine the prevalence of respiratory symptoms and related illnesses among the residents of Windhoek. To achieve this objective, descriptive analyses were performed for symptoms and related illnesses, and results are presented in Table 5. A high prevalence of respiratory symptoms was observed for cough (43%) and breathlessness (25%), with asthma recording the highest percentage for respiratory illness (11.2%). 

### 4.5. Association between Respiratory Disorders and PM Concentration, Location, and Confounding Variables

To further analyze for associations between respiratory disorders and PM concentration, location, and key confounding variables, a chi-square test of association was performed, and the results are presented in Table 6 and Table 7. Among all tested variables related to respiratory disorders, PM concentration was found to be only associated with episodes of cough and phlegm (*p* = 0.05). Moreover, a statistically significant association was observed between smoking (*p* = 0.02); history of occupational exposure to dust and chemicals for 1 year (*p* = 0.02), age group (*p* = 0.03), and episode of cough and phlegm for 3 months. While asthma (*p* = 0.01) and a grouped prevalence of any respiratory disease confirmed by doctor (*p* = 0.03) were found to be significantly associated with smoking alone (Table 6).

No significant association was observed between all types of respiratory health outcomes (symptoms and respiratory related illness) and house heating, vehicle movement near home, type of road, years at current home, total years respondent lived in Windhoek, distance from main road, or street and residence type of location (Table 7). A statistically significant association was observed between respondent type of energy used for cooking and cough symptoms (*p* = 0.03).

### 4.6. Relationship between Exposure Variables and Respiratory Disorders

The respiratory disorder variables that were found to be significantly associated with PM concentration, location, and confounding variables were fitted to analyze the relationship between respiratory disorders and exposure variables using unadjusted logistic regression models and the odds ratios are presented in Table 8. Association between PM concentration and episode of cough and phlegm had a 2.5 odds ratio for the high category and a 0.5 odds ratio for the medium category when compared to the low category. The association between type of energy used for cooking and cough had an odds ratio of 2.4. Analysis on the relationship between history of occupational exposure to dust and chemicals and history of smoking and episode of phlegm and cough indicates that respondents with a history of occupational exposure to dust or chemicals had a 0.2 odds ratio for experiencing episodes of cough and phlegm, while those with a history of smoking, regardless of current status, had a 0.25 odds ratio for episodes of cough and phlegm. The relationship between age group and episodes of cough and phlegm was further analyzed, with a high odds ratio of 4.4 observed for episodes of phlegm and cough among the age group of 31–40 years and those with a history of smoking had increased an odds ratio for asthma (5.9). 

## 5. Discussion

The study found high levels of PM concentration across all suburbs. The observed high levels of PM concentration could be attributed to the vehicle exhaust emission and re-suspension of road dust attributed to vehicle movements and the close proximity of respondents’ homes to the roads. This further explains the high PM concentration levels, across all suburbs, which were above the international recommended limits for residential areas. Other sources of observed high PM levels could be attributed to anthropogenic activities, of which its description is beyond the scope of this project. Moreover, the observed high PM levels might explain the high prevalence of cough, breathlessness as well as asthma, although no statistically significant association was observed. The observed high prevalence of asthma and to a certain extent bronchitis symptoms is in upkeep with findings of earlier studies [1]. 

The study found high prevalence levels of cough, breathlessness, and asthma, of which none were found to be associated with either the PM concentration or residence location. This is different from the results of previous studies which suggested that PM concentration was generally associated with acute and, to a certain extent, long term poor pulmonary related health outcomes, which was not associated with location, but only PM concentration being present across all suburbs [1,5]. The health effects attributed to PM exposure among Windhoek residents might be influenced by individual characteristics such as gender, age, socio-economic status, and health status and the effect might be further aggravated by time spent outdoors by individuals [5]. Among all of the assessed respiratory disorders, only an episode of phlegm and cough was found to be associated with PM concentration, while the cough symptom was observed to be associated with the type of energy used for cooking at home; this implies that regardless of one’s residential location, as long as one resides in WHK, the of risk of developing poor respiratory health outcomes is not entirely based on PM concentration, location, years at current home, or total years that one had lived in WHK. This could be explained by the observed high PM concentration levels across all suburbs, which could be further attributed to resident’s movement across the city as well as indoor related exposure to biomass fuel and environmental tobacco exposure [5]. Similarly, the observed association between PM and an episode of phlegm and cough supports earlier findings by earlier studies which associated episodic respiratory symptoms such as cough to PM concentration [1]. 

PM exposure causes irritation of the airway, with those exposed to higher PM concentrations reporting increased episodes of cough and with those exposed to gases reporting a higher incidence of wheezing than coughing, which is in agreement with this study [18]. 

In this study, the risk of experiencing cough symptoms was found to be associated with the type of energy used but not with PM concentration levels outside nor with indoor related pollutants. Although this is in agreement with many primary studies on indoor air quality and smoke exposure, it differs from PM related studies that reported an association between PM and cough [18].

Indoor air quality at home is primarily compromised by energy sources/type used for cooking, with those using solid fuels reporting a higher incidence of poor respiratory health, mainly because of exposure to smokes and other emissions resulting from the burning of fossil fuel [19]. 

The association observed in this study between cough and type of energy used is consistent with other studies. Such association may be due to the fact that the use of solid fuel or fossil fuel, such as paraffin, gas, or charcoal, reduces the indoor air quality, while increasing a user’s exposure to PM and other associated gases formed during the burning process [19]. Previous studies reported risk differences for health effects associated to PM concentration [20,21]. With respect to PM concentration and the observed high prevalence of respiratory outcomes, the study results are consistent with those conducted elsewhere [20,21,22]. This study did not find any association between poor respiratory health and gender, which could be due to the small sample size which may not have been sufficient to detect gender differences. 

The results, however, suggest that residential PM concentration alone is not responsible for poor respiratory health among WHK residents, but other factors such as age, smoking history, as well the history of occupational exposure to dust or chemicals may increase population vulnerability to poor respiratory health outcomes. The study found that WHK residents’ risk for poor respiratory health outcomes is multifactorial and may include factors such as environmental exposure to tobacco and poor indoor air quality both at home and at the work place. Furthermore, other studies reported a synergistic impaired effect between occupational exposure to dust or chemical pollutants and smoking on individual’s lungs, while smoking alone is proven to affect the lungs which contribute to the reduction of lung functional capacity and risk for poor respiratory health [23,24,25]. Similarly, an individual’s age is proven to increase his/her vulnerability to poor respiratory health outcomes mainly due to a weakened immune system and poor lung function, which render individuals to be susceptible to developing respiratory related symptoms and illness[24,25]. Thus the effect of other factors such as smoking, occupational exposure, and personal exposure to indoor pollutants needs to be quantified for the Namibian population to establish the overall country burden of respiratory diseases due to both indoor and outdoor pollutants. 

## 6. Limitation of the Study

Although the study is the first of its kind to be conducted in Windhoek, and that it provides snapshot types of evidence on both particle pollution levels in the city and prevalence of self-reported respiratory symptoms among the city residents, the study results need to be interpreted with the following limitations in mind: The study sample size was generally small and the method used to monitor particulate pollution levels does not account for PM size fraction and may not provide smaller PM concentrations. It is, however, ideal for repeated measurement for medium or long term trends in overall particle pollution in selected localities, which is in line with the objectives of this study. Also, the study may not provide conclusive evidence on the association between PM exposure and respiratory outcome, as those who might have been affected by PM exposure could have been in hospital and, therefore, may not have been reachable during the assessment time. Future studies should be conducted with increased sample size and improved long term PM monitoring methodology to provide trends on PM levels, which can be linked to respiratory related mortality and morbidity.

## 7. Conclusions

The study found high levels of PM concentration across all Windhoek suburbs, as well as high prevalence levels for cough, breathlessness, and asthma. The observed high prevalence level of respiratory symptoms was not found to be associated with any of the following: PM, individual location, house distance from road, or years that one had lived at current home or in WHK. Only episodes of cough and phlegm was found to be associated with PM, with those exposed to high levels observed to have an increased odds ratio of experiencing episodes of phlegm and cough. The type of energy used was observed to be a significant risk factor for cough symptoms among the residents, while history of smoking was a risk factor for asthma. Reduction of observed high PM levels and respiratory disorders requires enactment of legislation which addresses both the control and monitoring of PM related emissions at the point of generation. Moreover, health promotion interventions are needed to address risk factors such as smoking and use of biomass fuel which were identified as factors that increase population risk of poor respiratory health outcomes. 

## Figures and Tables

**Table 1:** Participants’ demographic information.

Variables		Frequency and Percentages
Age Group (Years)	18–30	56 (52.3)
	31–40	20 (18.7)
	≥41	21 (19.6)
Gender	Male	55 (51.4)
	Female	52 (48.6)
Marital Status	Married	19 (17.8)
	Widowed	1 (0.9)
	Divorced	2 (1.9)
	Separated	5 (4.7)
	Never Married	79 (73.8)
Cigarette Smoking	Ex-Smoker	8 (7.5)
	Current Smoker	8 (7.5)
	Never Smoker	91 (85)
Distance from Road Category (m)	≤10	35 (32.7)
	10.1–90	33 (30.8)
	≥91	39 (36.4)
Years at Current Home	≤2	36 (33.6)
	3–6	36 (33.6)
	≥7	35 (32.7)
Total Years Lived in WHK (Windhoek)	2–4	31 (29.0)
	5–14	39 (36.4)
	≥15	37 (34.6)
Employment Status	Employed	56 (52.3)
	Student	21 (19.6)
	Unemployed	28 (26.2)

**Table 2:** Relative frequency of environmental related exposure to Particulate Matter (PM).

Variables		*N* (%)
Type of Road Passing by the House	Gravel Road	24 (22.4)
	Tar Road	83 (77.6)
Occupational Dust Exposure > 1 Year	Yes	12 (11)
	No	95 (89)
Type of Fuel Used for Cooking	Solid Fuel	1 (0.9)
	Gas	32 (30)
	Electricity	68 (63.5)
	Paraffin	6 (5.6)
Vehicle Movement near House	Constantly	47 (43.9)
	Frequently	42 (39.3)
	Seldom	17 (15.9)
	Never	1 (0.9)
Finds Dust on Indoor Surfaces	Constantly	45 (42.1)
	Frequently	36 (33.6)
	Seldom	20 (18.7)
	Never	6 (5.6)

**Table 3:** PM concentration in mg/m^2^/day for sampling periods 1–3. (*N* = 45).

Suburb	Residence Type	PM in mg/m^2^/day for Sampling Period 1	PM in mg/m^2^/day for Sampling Period 2	PM in mg/m^2^/day for Sampling Period 3	Mean PM in mg/m^2^/day for the Entire Sampling Period
Windhoek West	Medium Density	1745.93 **	789.61 **		1267.8
Wanahenda	High Density	2170.1 **	1673.7 **	1598.14 **	1814.0
Onlympia	Low Density	781.1	533.33 ^†^	444.31 ^†^	586.2
Okurganva	High Density	2128.63 **	3030.7 **	-	2579.7
Khomasdal	Medium Density	2787.11 **	1606.05 **	-	2196.6
Havanna	High Density	2453.56 **	1777.44 **	-	2115.5
Hochland	Low Density	1171.29 **	902.87 **	-	1037.1
Hakahana	Medium Density	3387.43 **	3102.02 **	-	3244.7
Freedom Square	Medium Density	-	1039.15 **	-	1039.2
Soweto	Medium Density	-	2285.04 **	2128.76 **	2206.9
Golgata	Medium Density	-	2079.07 **	2350.85 **	2215.0
Freedomland	Medium Density	-	2195.5 **	1198.6 **	1697.1
Ombili	Medium Density	-	2015.43 **	1928.22 **	1971.8
Ludwig/Olyimpia	Low Density	690.48 **	427.13 ^†^	-	558.8
Kahandja Park	High Density	-	2068.06 **	2767.11 **	2417.6

** = Exceeded Residential Limit Value, ^†^ = Within Limit, - = Missing Data.

**Table 4:** Variance in PM in concentration.

(I) Residence Type	(J) Residence Type	Mean Difference (I-J)	Standard Error	Significance	95% Confidence Interval (95% CI)	
					Lower Bound	Upper Bound
Low Density	Medium	−576.72000	533.99306	0.301	−1740.1909	586.7509
	High	−1466.06200 *	385.06787	0.002	−2305.0528	−627.0712
Medium	Low Density	576.72000	533.99306	0.301	−586.7509	1740.1909
	High	−889.34200	453.10813	0.073	−1876.5798	97.8958
High	Low Density	1466.06200 *	385.06787	0.002	627.0712	2305.0528
	Medium	889.34200	453.10813	0.073	−97.8958	1876.5798

* The mean difference is significant at the 0.05 level.

**Table 5:** Prevalence of respiratory health outcomes (*N* = 107).

Respiratory Symptoms	*n* (%)
Usual Cough	46 (43)
Usual Phlegm	19 (18)
Episode of Cough and Phlegm	18 (16)
Breathlessness	27 (25)
Wheezing	18 (16)
Bronchitis	5 (4.7)
Emphysema	1 (0.9)
Asthma	12 (11.2)
Tuberculosis	3 (2.8)
Chest Illness	2 (1.9)
Any Respiratory Symptoms	61 (57)
Any Respiratory Diseases	17 (15.9)

**Table 6:** Chi-square test of association between respiratory disorders, PM concentration, and confounding variables.

Variables	Smoking		History of Occupational Exposure		Age Group		Tuberculosis		PM Concentration	
Respiratory Symptoms	*df*	*p*-Value	*df*	*p*-Value	*df*	*p*-Value	*df*	*p*-Value	*df*	*p*-Value
Usual Cough	1	0.2	1	0.9	4	0.7	1	0.07	1	0.07
Usual Phlegm	2	0.7	2	0.3	8	0.2	1	0.08	1	0.6
Episode of Cough and Phlegm	1	0.02 *	1	0.02 *	4	0.03 *	**1**	**0.4**	**1**	0.05 *
Breathlessness	1	0.07	1	0.4	4	0.3	1	0.2	1	0.7
Wheezing	1	0.5	1	0.7	4	0.8	1	0.4	1	0.08
Bronchitis	1	0.6	1	0.5	4	0.2	1	0.1	1	0.3
Chronic Bronchitis		0.3		0.8	4	0.8	1	0.03	1	0.7
Asthma	1	0.01 *	1	0.6	4	0.2	1	0.03	1	0.6
Tuberculosis	1	0.3	1	0.7	4	0.8	-	-	-	0.4
Chest Illness	1	0.7	1	0.8	4	0.8	1	0.05	1	0.1
Any Respiratory Symptoms	1	0.6	1	0.6	4	0.3	1	0.2	1	0.3
Any Respiratory Diseases	1	0.03 *	1	0.4	4	0.5	1	0.004	1	0.9

*df* = degree of freedom, * *p*-Value < 0.05.

**Table 7:** Association between respiratory disorders and PM concentration and location.

Variables	Type of Energy Used for Cooking		House Heating		Vehicle Movement near Home		Indoor Dust		Types of Road		Years at Current Home		Total Years in Windhoek		House Distance from Main Road/Street		Location or Place of Residence	
	*df*	*p*-Value	*df*	*p*-Value	*df*	*p*-Value	*df*	*p*-Value	*df*	*p*-Value	*df*	*p*-Value	*df*	*p*-Value	*df*	*p*-Value	*df*	*p*-Value
Cough	1	0.03 *	2	0.2	1	0.5	1	0.08	1	0.4	2	1.0	2	0.1	2	0.8	2	0.2
Usual Phlegm	2	0.6	4	0.9	2	0.8	2	0.3	2	0.8	4	0.4	4	0.5	4	0.5	4	1.0
Episode of Cough and Phlegm More than 3 Months	1	0.2	2	0.8	1	0.5	1	0.4	1	0.2	2	0.5	2	0.4	2	0.7	2	0.4
Breathlessness	1	0.6	2	0.6	1	0.4	1	0.9	1	1.0	2	0.3	2	0.2	2	0.2	2	0.6
Wheezing	1	0.4	2	0.07	1	1.0	1	0.3	1	0.6	2	0.5	2	0.4	2	0.4	2	0.2
Attack of Bronchitis	1	1.0	2	0.9	1	0.6	1	0.3	1	0.7	2	0.2	2	0.8	2	0.3	2	0.3
Chronic Bronchitis	1	1.0	2	0.5	1	1.0	1	0.6	1	0.4	2	0.1	2	0.6	2	0.2	2	0.4
Emphysema	1	1.0	2	0.4	1	1.0	1	0.8	1	0.8	2	0.4	2	0.4	2	0.3	2	0.08
Asthma	1	0.7	2	0.9	1	0.7	1	0.6	1	0.5	2	0.1	2	0.6	2	0.6	2	0.7
Tuberculosis	1	0.2	2	0.9	1	1.0	1	0.4	1	0.5	2	0.4	2	0.07	2	0.4	2	0.06
Any Other Chest Illness Confirmed by the Doctor	1	0.3	2	0.5	1	1.0	1	0.6	1	0.6	2	0.6	2	0.6	2	0.6	2	0.4
Respiratory Symptoms (Grouped Symptoms)	1	0.1	1	0.5	1	0.3	1	0.1	1	0.9	2	0.8	2	0.1	2	0.4	2	0.08
Doctor Diagnosis of Respiratory Diseases	1	0.6	1	0.5	1	0.6	1	0.4	1	0.4	2	0.3	2	0.5	2	0.9	2	0.7

* *p*-Value < 0.05.

**Table 8:** Odd ratios (OR) of selected respiratory disorders from unadjusted logistic regression models.

Variables		Usual Cough	Cough for More than 3 Months	Usual Phlegm	Episodes of Phlegm and Cough	Shortness of Breath	Wheezy Chest	Asthma	Any Symptom	Doctor Diagnosed Diseases
		OR (95% CI)	OR (95% CI)	OR (95% CI)	OR (95% CI)	OR (95% CI)	OR (95% CI)	OR (95% CI)	OR (95% CI)	OR (95% CI)
PM Concentration	Low PM	-	-	-	1	-	-	-	-	-
	Medium PM	-	-	-	0.5, 0.1–2.3	-	-	-	-	-
	High PM	-	-	-	2.5, 0.8–8.0	-	-	-	-	-
Never Smokers		-	-	-	1	-	-	1	-	1
Ever-Smoker		-	-	-	0.25, 0.01–0.83	-	-	5.9, 1.6–22.2	-	0.14, 0.04–0.48
Age Group (Years)	≤30	-	-	-	1	-	-	-	-	-
	31–40	-	-	-	4.4, 1.2–16.5	-	-	-	-	-
	≥41	-	-	-	4.1, 1.1–15.3	-	-	-	-	-
Not Occupationally Exposed		-	-	-	1	-	-	-	-	-
Occupationally Exposed		-	-	-	0.2, 0.1–0.8	-	-	-	-	-
Type of Energy Used	Electricity	1	-	-	-	-	-	-	-	-
	Biomass Fuel and Others	2.4, 1.1–5.3	-	-	-	-	-	-	-	-
